# Efficacy of vitamin D supplement in children with nonalcoholic fatty liver disease: A protocol for systematic review and meta-analysis

**DOI:** 10.1097/MD.0000000000020960

**Published:** 2020-07-31

**Authors:** Jie Liu, Tingting Wang, Junmin Chen, Yanli Zhang, Xiaoqin Yin, Xueqin Fan, Qiu Chen

**Affiliations:** Hospital of Chengdu University of Traditional Chinese Medicine, Chengdu, P.R.

**Keywords:** nonalcoholic fatty liver disease, protocol, systematic review and meta-analysis, vitamin D

## Abstract

**Background:**

The number of children with nonalcoholic fatty liver disease (NAFLD) is proliferating. However, currently, there are no drugs approved for the management of NAFLD. There have been some studies on vitamin D and NAFLD in children. However, the effectiveness of vitamin D in children with NAFLD has not been systematically evaluated. The objective of this systematic review will be to evaluate the effectiveness of vitamin D in children with NAFLD.

**Methods:**

We will search through PubMed, Google Scholar, Cochrane Library, Web of Science, and the ClinicalTrials.gov website without restriction on publishing status. To supplement our search strategy, we will scan the reference lists of the identified studies for detailed evaluation for additional possible eligible studies, and we will also search conference proceedings related to this topic. All databases were searched from inception to present. Any clinical randomized controlled trials related to vitamin D supplement for treating NAFLD (simple steatosis/NAFL and NASH) in children will be included. NAFLD had to be diagnosed by liver histology, imaging (ultrasound, computer tomography, magnetic resonance imaging). The primary outcomes will be changes in liver fibrosis and liver enzymes. The variations in serum vitamin D level, BMI, insulin levels, lipid profiles, liver fat content will also be assessed. All statistical analyses will be carried out using RevMan, version 5.3, Copenhagen: The Nordic Cochrane Centre, The Cochrane Collaboration, 2014.

**Results:**

This study will provide a comprehensive high-quality synthesis to evaluate the effectiveness of vitamin D supplementation in children with NAFLD.

**Conclusion:**

This systematic review and meta-analysis will provide evidence to judge whether vitamin D supplementation is an effective intervention for children with NAFLD.

**Registration number:**

INPLASY202050049.

## 1 Introduction

Nonalcoholic fatty liver disease (NAFLD) is a group of clinical-pathological syndromes ranging from benign steatosis to nonalcoholic steatohepatitis (NASH), which may eventually develop into acute liver injury, including cirrhosis, and hepatocellular carcinoma (HCC).^[1,2]^ The occurrence of the disease is closely related to multiple factors such as overnutrition, obesity, insulin resistance, metabolic syndrome, oxidative stress, and cytokines.^[3]^ NAFLD is now considered an independent risk factor for cardiovascular disease.^[4]^ In fact, NAFLD is a risk factor for many diseases. Following up patients with NAFLD for 5 to 10 years, the risk of type 2 diabetes mellitus (T2DM) increased by 1.86 times (95%CI 1.76–1.95), the risk of metabolic syndrome (MetS) increased by 3.22 times (95%CI 3.05–3.41), and the risk of NAFLD increased by 1.64 times (95%CI 1.26–2.13).^[5,6]^ In addition, NAFLD, especially NASH, is associated with a high incidence of chronic diseases such as osteoporosis, chronic kidney disease, colorectal tumors, and breast cancer.^[7–9]^ Currently, the prevalence of NAFLD is about 25% in the global and Asian populations.^[10,11]^ NAFLD is not only prevalent in adults, but also very common in children and adolescents.

Children's NAFLD is a clinicopathological syndrome of chronic fatty liver degeneration involving more than 5% of liver cells in children and adolescents under the age of 18 years, excluding alcohol consumption and other definite pathogenic factors leading to chronic fatty deposition in the liver. The spectrum of diseases included nonalcoholic fatty liver (NAFL), NASH and its associated liver fibrosis and cirrhosis.^[12]^ In America, the prevalence of NAFLD children is 3% to 11%.^[13]^ The prevalence of NAFLD in Asian and Chinese children was 6.3% and 3.4% respectively.^[14]^ The prevalence of NAFLD was significantly increased in obese and overweight children, increased to 50% to 80%, and became the common reason for chronic liver disease in children.^[15]^ However, to date, no effective medical interventions exist that completely reverse the disease other than lifestyle changes, dietary alterations. It can be difficult for children to improve their NAFLD status by making lifestyle changes and exercising. The study had found that surgical weight loss can improve dyslipidemia and insulin resistance in children with NAFLD and obesity.^[16]^ However, there are few relevant studies and insufficient experience to evaluate its advantages. Therefore, drugs with definite efficacy are needed to treat NAFLD in children.

Vitamin D is a fat-soluble vitamin that is synthesized naturally in the skin during exposure to solar radiation, specifically ultraviolet-B. It is a steroid hormone, which is involved in immune, inflammation, lipid metabolism, and other pathophysiological reactions in vivo, affecting cell proliferation, differentiation and apoptosis.^[17]^ Vitamin D is closely related to the occurrence and development of liver diseases. Vitamin D is involved in the regulation of immune response, virus replication, liver fibrosis, lipid metabolism, liver cancer, and other aspects.^[18]^ In patients with healthy liver enzymes, vitamin D deficiency is associated with the incidence and severity of NAFLD.^[19,20]^ Study has shown that vitamin D can reduce serum lipid concentration, reduce the expression of genes related to liver fat production, improve oxidative stress, and weaken liver steatosis in rats on a high-fat diet.^[21]^ And vitamin D lack, can activate the toll-like receptors, increase obesity rats of NAFLD.^[22]^ Similarly, it has been found in clinical studies that VD deficiency is more common in obese people.^[23,24]^ Study has shown low vitamin D levels are also connected with liver steatosis, necroinflammation, and fibrosis in patients with NAFLD.^[25]^ Studies in animal models have shown that vitamin D deficiency aggravates the histology of NAFLD.^[26,27]^ Besides a meta-analysis of the adult NAFLD study showed an association between reduced levels of vitamin D and NAFLD,^[28]^ and studies have shown that low levels of vitamin D are associated with the severity of steatosis.^[19]^ And, a meta-analysis showed that vitamin D deficiency was prevalent in children and adolescents with NAFLD, and that vitamin D may be associated with progression and severity of NAFLD.^[29]^ However, there has been no a large sample size clinical trials to confirm the effect of vitamin D on NAFLD in children. Thus we conducted a meta-analysis to reveal the efficacy of vitamin D supplement on children with NAFLD.

## 2 Methods

This protocol has been registered on the INPLASY website with registration number INPLASY202050049. (https://inplasy.com/inplasy-2020-5-0049/). The protocol follows the Preferred Reporting Items for Systematic Reviews and Meta-Analyses Protocols (PRISMA-P) statement guidelines.^[30,31]^

### 2.1 Eligibility criteria

#### 2.1.1 Types of studies

All RCTs without restrictions on publication status will be included. Case report, case series, cross-sectional, case controls, retrospective studies, and observational studies will be excluded.

#### 2.1.2 Types of participants

Patients were eligible for the study if they were children (age <18 years) populations of any sex or ethnicity, NAFLD (simple steatosis/NAFL and NASH). NAFLD had to be diagnosed by liver histology, imaging (ultrasound, computer tomography, magnetic resonance imaging). Moreover, in all children, significant alcohol intake and other known causes of liver disease, and the use of drugs known to induce fatty liver, were also excluded.

#### 2.1.3 Types of interventions

##### 2.1.3.1 Experimental interventions

This meta-analysis will include the RCTs of vitamin D supplementation regardless of dose and frequency. Trials with a minimum treatment duration of at least 4 weeks will be covered.

##### 2.1.3.2 Control interventions

Comparison interventions include placebo control, no therapy, exercise intervention, diet intervention, or active pharmacological treatment.

#### 2.1.4 Types of outcome measures

##### 2.1.4.1 Primary outcomes

Primary outcomes include the effects of vitamin D supplement on liver fibrosis and liver enzymes.

##### 2.1.4.2 Secondary outcomes

Secondary outcomes include the changes of serum vitamin D level, BMI, insulin levels, lipid profiles, liver fat content after treatment of vitamin D supplement.

### 2.2 Search methods for the identification of studies

#### 2.2.1 Electronic searches

Relevant studies were identified in the following electronic databases: PubMed, Google Scholar, Cochrane Library, Web of Science, and ClinicalTrials.gov. Clinical trial registries were also searched for unpublished trials. All databases were searched from inception to present. We will only include studies published in English and Chinese. The search strategy on PubMed MEDLINE is listed in Table 1.

**Table 1:**
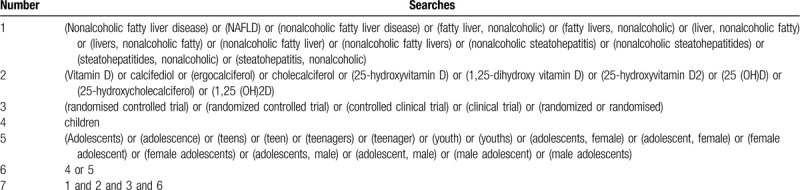
PubMed Medline search strategy.

#### 2.2.2 Other resources

To identify additional trials, we will hand search for the bibliographies of all included studies, as well as any reviews on the topic. We will also search for conference proceedings related to this topic.

### 2.3 Data collection and analysis

#### 2.3.1 Selection of studies

**Figure 1:**
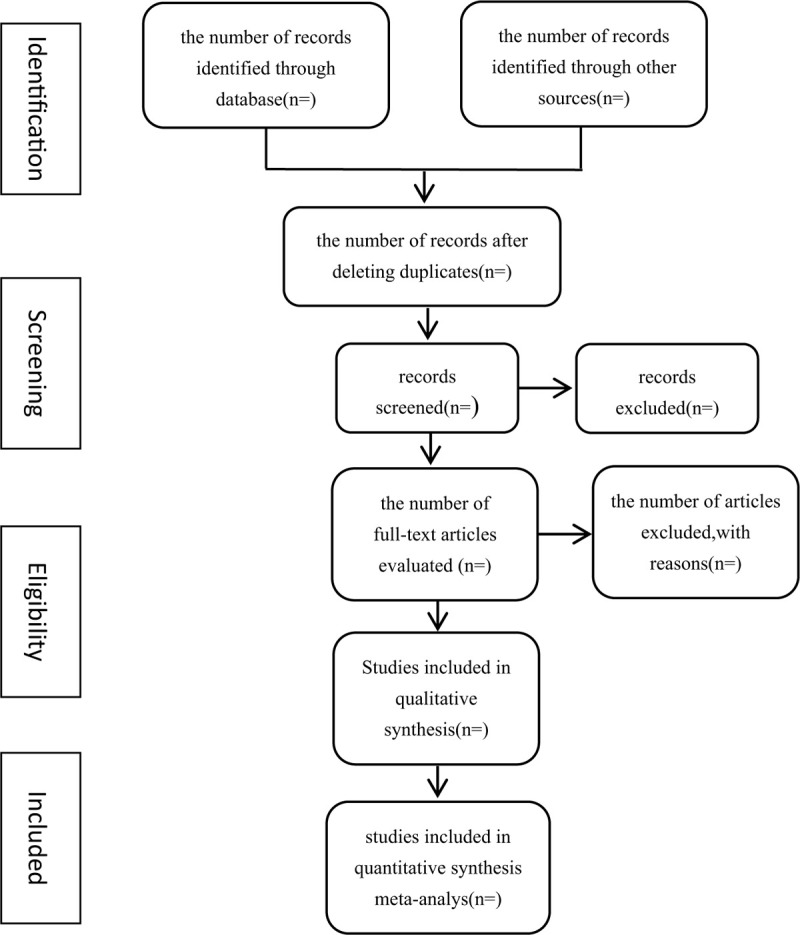
Flow chart of the study selection.

Two review authors will independently screen the titles and abstracts of all retrieved studies and select all potentially relevant references. At this stage of the screening process, reviewers are told to relax their restrictions. If there was insufficient information to conclude that a given citation should be excluded certainly, the citation was retained and included in the full-text screening stage. All records will be managed with Endnote X9 in a separate database. The 2 reviewers will then independently read the full texts and select studies to be included based on our predetermined inclusion criteria. In the case of unclear information or missing data, we will contact the original authors for clarification. The studies that do not fulfill the inclusion criteria will be excluded and listed with reasons for their exclusion. Any disagreements will be resolved by discussion between the 2 authors and the more experienced author for arbitration when necessary. The study flow diagram is shown in Figure 1.

#### 2.3.2 Data extraction and management

Data for the trials will be extracted independently by 2 review authors using a standard form. The following information will be included:

1) general information on the publications (title, authors, country, journal name, year of publication, etc).
2) Study methods and characteristics (sample size, design, NAFLD assay methods, randomization, blinding, inclusion/exclusion criteria, and withdrawals/dropouts).
3) Participants (number of participants, age, gender, racial ancestry, disease severity, clinical, and laboratory parameters).
4) Intervention (dose and type of vitamin D, frequency, duration).
5) Control (no treatment, placebo therapy, exercise intervention, diet intervention, other active treatment).
6) Outcomes (outcome data for the main outcomes and additional outcomes of interest).

Extracted data will be compared by 2 review authors for completeness and accuracy and double-checked by another review author if necessary. The original authors will be contacted in case of missing data. Disagreements will be resolved by discussion between the 2 authors, and further disputes will be arbitrated by the third author.

#### 2.3.3 Risk of bias assessment

The risk of bias in the included studies will be assessed independently by 2 authors and presented at a risk of bias table. The decision will be based on the Cochrane collaboration's tools^[32]^ to assess the areas and criteria for the risk of bias. We will evaluate the following domains for risk of bias: sequence generation, allocation sequence concealment, blinding of participants and personnel and outcome assessors, incomplete outcome data, selective outcome reporting, and other sources of bias. The assessments will be classified into 3 levels: low risk, high risk, and unclear risk. If there is disagreement about bias, the decision is discussed through 2 reviewers or with a third reviewer.

#### 2.3.4 Assessment of heterogeneity

We will use the I^2^ statistic for quantifying inconsistencies among the included studies. When the I^2^ value is less than 50%, we believe that there is no heterogeneity in this study. If the value of I^2^ is 50% or more, it indicates a large degree of inconsistency. If we identify substantial heterogeneity, we will report it and explore possible causes using subgroup analyses.

#### 2.3.5 Measures of treatment effect

For continuous data, the mean difference (MD) or standardized mean difference (SMD) will be used to measure the treatment effect with 95% CI. For dichotomous data, treatment effects are presented as a risk ratio (RR) with 95% CI.

#### 2.3.6 Dealing with missing data

For each included study, missing data will be gathered by contacting the study author. If the relevant data is not available after contacting the author, the pertinent missing data will not be included in the analysis. And a sensitivity analysis will be used to determine whether these missing data affect the results of the meta-analysis. If necessary, we will describe the potential impact of the lack of data on the outcome of the review in the “Discussion” section.

#### 2.3.7 Data synthesis

Data synthesis will be performed with Cochrane Review Manager (V.5.3) when a meta-analysis is allowed. If the I^2^ test is less than 50%, the fixed-effects model will be used for data synthesis. If the I^2^ test is higher than 50%, the random-effects model will be conducted for data synthesis. If clinical and methodological heterogeneity is present, a subgroup analysis will be performed to investigate possible sources of statistical heterogeneity. When meta-analysis is not available, we will conduct a systematic narrative synthesis providing information to summarise and explain the characteristics and findings of the included studies.

#### 2.3.8 Subgroup analysis

We plan to carry out following subgroup analyses if sufficient comparable studies are identified.

1) Comparison between dose and type of vitamin D, frequency, duration,
2) Comparison between different baseline Serum vitamin D levels.
3) Comparison between NAFL and NASH.

#### 2.3.9 Sensitivity analysis

In order to investigate the stability of the results, we will conduct a sensitivity analysis for the outcomes by omitting each of the RCT, or excluding the RCTs with a high risk of bias, or excluding the RCTs with missing data. In this way, we will be able to assess the impact of individual studies on the overall results and whether the results are reliable. The conclusions will be compared and discussed according to the analysis results.

#### 2.3.10 Assessment of reporting biases

If more than 10 studies are included in the meta-analysis, a funnel plot is created and examined to explore possible research biases and carefully interpret the results.

#### 2.3.11 Ethics and dissemination

No ethical approval will be required because we will use data from previously published studies in which informed consent was obtained by the primary investigators. We will publish our results in a peer-reviewed journal.

## 3 DISCUSSION

NAFLD is the most common cause of liver disease worldwide. With the improvement of living standards, the number of obese children is increasing, and the detection rate of NAFLD is also on the rise. However, there is no recognized cure. There has been a lot of research on vitamin D and non-alcoholic fatty liver disease. However, there are differences between studies on the effect of vitamin D in children with NAFLD. At present, there is no systematic and comprehensive summary of the existing clinical evidence to assess the efficacy of vitamin D on NAFLD in children. In this study, we will conduct this systematic review and meta-analysis to provide more evidence-based medical support for the clinical use of vitamin D supplement in children with NAFLD.

However, the proposed systematic review has some potential limitations. There may be a language bias in this study, as we will only include studies published in English and Chinese, which means that some related studies in other languages may be missed.

## Author contributions

**Conceptualization:** Jie Liu

**Data curation:** Jie Liu, Tingting Wang

**Formal analysis:** Tingting Wang, Xiaoqin Yin, Xueqin Fan

**Funding acquisition:** Qiu Chen

**Methodology:** Jie Liu, Xueqin Fan, Yanli Zhang

**Project administration:** Qiu Chen

**Resources:** Jie Liu, Junmin Chen, Xiaoqin Yin

**Software:** Tingting Wang, Junmin Chen, Yanli Zhang

**Supervision:** Qiu Chen

**Writing – original draft:** Jie Liu, Tingting Wang

**Writing – review & editing:** Qiu Chen
